# In Comparison to PSA, Interim Ga-68-PSMA PET/CT Response Evaluation Based on Modified RECIST 1.1 After 2^nd^ Cycle Is Better Predictor of Overall Survival of Prostate Cancer Patients Treated With ^177^Lu-PSMA

**DOI:** 10.3389/fonc.2021.578093

**Published:** 2021-03-17

**Authors:** Vikas Prasad, Kai Huang, Sonal Prasad, Marcus R. Makowski, Norbert Czech, Winfried Brenner

**Affiliations:** ^1^Department of Nuclear Medicine, Charité - Universitätsmedizin Berlin, Berlin, Germany; ^2^Department of Nuclear Medicine, University Hospital Ulm, Ulm, Germany; ^3^Berlin Experimental Radionuclide Imaging Center, Charité – Universitätsmedizin Berlin, Berlin, Germany; ^4^Department of Radiology, Charité - Universitätsmedizin Berlin, Berlin, Germany; ^5^Center for Nuclear Medicine and PET/CT, Bremen, Germany; ^6^German Cancer Consortium (DKTK), Partner Site Berlin, Berlin, Germany

**Keywords:** radioligand therapy, Lu-177-PSMA, interim staging, Ga-68-PSMA PET/CT, PSA response

## Abstract

**Background:**

Prostate-specific membrane antigen (PSMA) targeting radioligands have transformed treatment of prostate cancer. Radioligand therapy (RLT) with ^177^Lu-PSMA in metastasized castration resistant prostate cancer (mCRPC) achieves objective response and disease stabilization in roughly two third of patients, whereas one third of patients progress. This study was performed to assess the role of interim PSMA PET/CT after the 2^nd^ cycle of RLT for early prediction of overall survival in patients undergoing RLT with ^177^Lu-PSMA.

**Methods:**

38 mCRPC patients (68.9 ± 8.1 y) treated with at least two cycles of RLT at 8 week intervals and interim ^68^Ga-PSMA PET/CT (PET) at 8–10 weeks after the 2^nd^ cycle of RLT were included in this study. Prostate-specific antigen (PSA) response was evaluated according to the Prostate Cancer Working Group 3 criteria. Radiographic response assessment of soft tissue, lymph node, and bone lesions was performed according to RECIST 1.1 including the PET component. Patients’ data were collected for follow-up from the local Comprehensive Cancer Center Register.

**Results:**

Median follow-up was 19.7 months (4.7–45.3). PSA response after the 2^nd^ therapy cycle showed partial remission (PR) in 23.7%, stable disease (SD) in 50%, and progressive disease (PD) in 26.3% of patients. In comparison, 52.6, 23.7, and 23.7% of patients showed PR, SD, and PD respectively on PET/CT. The strength of agreement between PSA response and PET/CT response criteria was only fair (kappa 0.346). Median overall survival (OS) was 22.5 months (95% CI: 15.8–29.2). Median OS stratified to PSA/PET response was 25.6/25.6 months for PR, 21.7/30.6 months for SD and 19.4/13.1 months for PD (p = 0.496 for PSA and 0.013 for PET/CT response).

**Conclusions:**

Interim PSMA PET/CT based response evaluation at 8–10 weeks after the 2^nd^ cycle of RLT is predictive of overall survival and PD in patients treated with ^177^Lu-PSMA. On the contrary, PSA appears to have only limited predictive value.

## Introduction

Prostate cancer (PCa) is one of the most common cancers and the second most frequent cause of cancer related mortality in the United States (1). Despite advancement in the therapeutic armamentarium, the 5-year survival rate is approximately three times higher in patients with locally advanced PCa in comparison to metastasized PCa (1). This highlights the need for development of novel treatment options and improvement of existing ones for metastasized PCa. In addition, considering the number of different treatment options and their toxicity, standardization of the sequences of treatment regimens and defining subgroups of patients with poor outcomes upfront are an absolute necessity. One of the ways to achieve this is by using specific targets on PCa for diagnosis and treatment.

Prostate specific membrane antigen (PSMA) is over-expressed in PCa, both on the primary tumor as well as in metastases (2). Labeled with radionuclides, *e.g.* ^68^Ga or ^18^F, PSMA PET/CT has high diagnostic performance for staging and restaging of PCa patients (3–5). Furthermore, PSMA imaging allows visualization and quantification of target expression for radionuclide treatment with ^177^Lu-PSMA (6, 7).

Several previous studies have shown safety and efficacy of radioligand therapy (RLT) with ^177^Lu-PSMA in metastasized castrate resistant prostate cancer (mCRPC) patients (8, 9). Although there are different protocols for RLT, majority of the guidelines recommend treatment with four therapy cycles (8, 10, 11). RLT achieves objective response and disease stabilization in roughly two third of patients, whereas one third of patients progress. Unfortunately, even now there are not much data to rely on reliable biomarkers for prediction of response to RLT. Some potential predictors for response have been reported such as elevated alkaline phosphatase or lactate dehydrogenase, presence of visceral metastases, higher intensity of tumor uptake, previous chemotherapy, *etc*. (8, 12, 13). However, none of these parameters are implemented in the response assessment of PCa. Literature on PSA based response criteria as predictor for overall survival after RLT is lacking. Similarly, data is sparse on the predictive role of interim PSMA PET/CT after 2^nd^ RLT.

In our study, we assessed the role of interim PSMA PET/CT (defined as PET/CT performed 8–10 weeks after the 2^nd^ cycle of treatment) for prediction of overall survival in patients who received at least two cycles of ^177^Lu-PSMA 617.

## Methods

### Patient Population

Between 2015 and 2017, a total of 63 patients were treated with RLT with ^177^Lu-PSMA 617. Only patients (n = 38) who were investigated with interim PSMA PET/CT after two therapy cycles were included for the analyses. Data of thirty-eight patients (68.9 ± 8.1 y) with histologically confirmed PCa treated with at least two cycles of RLT at 8 weeks interval were retrospectively analyzed. Response assessment by PET/CT was performed at 8–10 weeks after the 2^nd^ therapy cycle. All patients analyzed in this study gave written informed consent for treatment and analyses of their clinical data, and the study was approved by the local institutional ethics committee (EA2/177/17). RLT with ^177^Lu-PSMA and imaging with ^68^Ga-PSMA were both performed under national regulations of compassionate use of a non-approved drug according to AMG §13.2b (German Medicinal Product §13.2b) (14, 15). All patients had a metastasized castrate resistant prostate cancer progressive disease and showed PSMA expression on ^68^Ga-PSMA PET/CT. The decision for ^177^Lu-PSMA therapy was taken in an interdisciplinary setting. Decision to continue or discontinue RLT after interim staging after two RLT cycles was also taken in an interdisciplinary setting.

### Preparation and Administration of ^177^Lu-PSMA

For radiolabeling, PSMA-617 was obtained from ABX Advanced Biochemical Compounds (Radeberg, Germany), and ^177^Lu was obtained from ITM (Munich, Germany). The process of radiolabeling has been described in detail elsewhere (5). According to the national regulations, quality control procedures were performed by an experienced radiochemist, and the final decision regarding the appropriateness of radiolabeling and the decision for application of the ^177^Lu-PSMA were taken by board-certified nuclear medicine physicians, all with more than 10 years of experience in targeted radionuclide therapy. Radiochemical purity was more than 99% in all cases using, TLC using Wallac Wizard Gamma Counter and radio- HPLC system (Knauer Azura).

^177^Lu-PSMA-617 was administered according to the consensus guideline of the German Nuclear Medicine Society (10). Briefly, patients were hydrated with 1.5 l of NaCl infusion 30 min before the start of the infusion of the radiopharmaceutical. Patients received ^177^Lu-PSMA-617 as an i.v. infusion over 10–15 min. Blood pressure and vital signs were monitored at close intervals for 1 h after the injection.

### Efficacy and Response

Biochemical response was assessed using PSA values determined immediately prior to the first cycle and 8–10 weeks after the second treatment cycle. According to the Prostate Cancer Work Group 3 criteria, a PSA decline ≥50% was considered as partial response, and a PSA rise ≥25% as progressive disease, and values in between were considered as stable disease (16).

### Image Based Response Assessment

PSMA PET/CT (diagnostic CT facultative with iodine contrast media) was performed as already described (5). Baseline ^68^Ga-PSMA PET/CT was performed within 4 weeks prior to the first cycle RLT with ^177^Lu-PSMA. Response assessment by PET/CT was performed at 8–10 weeks after the 2^nd^ cycle RLT with ^177^Lu-PSMA. Further imaging was performed at subsequent 3 months intervals or earlier based on the clinical needs/biochemical progression.

Use of RECIST for evaluation of response to systemic therapy in prostate cancer has some major limitations due to the common presence of osteoblastic skeletal lesions which are considered non-measureable. To overcome this issue, analog to RECIST 1.1, we incorporated PET information to evaluate bone lesions. In RECIST 1.1 Bone scan, PET scan or plain films are not considered adequate imaging techniques to measure bone lesions. However, both bone scan and PET scan have been suggested as a possible means to confirm the presence or disappearance of bone lesions. 2-deoxy-2-[^18^F]-fluoro-D-glucose (FDG)-PET can be used for detection of possible new disease in RECIST 1.1.

With this background, we defined response on ^68^Ga-PSMA PET/CT, hitherto named as RadioLigand Therapy Response Evaluation Criteria for Prostate Cancer or RLT-REC-PCA, as follows:

- Complete Remission (CR): no lesion seen on CT and PET
- Partial Remission (PR): decrease of more than 30% of the sum of target lesions’ diameter (for lymph nodes, liver, and lung metastases) and no evidence of new visceral and bone lesions on ^68^Ga-PSMA PET or on CT
- Progressive Disease (PD): more than 20% increase in the sum of the target lesions and/or at least >5 mm increase in the target lesion, and/or evidence of new bone lesions or evidence of new lymph node or soft tissue lesions on PET or CT
- Stable Disease: neither CR, PR, or PD.

All the images were analyzed by two board certified nuclear medicine physicians and one radiologist. Any discrepancies between PET and CT were resolved using best valuable comparator comprising of clinical, imaging, and biochemical follow-up (17). As interobserver correlation with PSMA PET/CT is very high, any discrepancies were, in addition to using the best valuable comparator, also solved by taking a common consensus during reporting.

### Statistics

IBM SPSS version 24 was used for statistical analysis. Data are presented as mean, median, and ranges or as frequencies. Kaplan–Meier curve was used to evaluate differences in OS between patients with PSA/PSMA PET/CT PD and those with non-PD, or PR, and SD. All tests were two-sided. Cohens Kappa test was performed to calculate the strength of agreement between PSA and PSMA PET/CT based response assessment. Fisher exact test was performed to rule out non-random association between two categorical variables. P < 0.05 was taken as significant.

## Results

Patients were treated with 5.5 ± 1 GBq RLT/cycle; median of three (range 2–5) cycles per patient at 8-week intervals was performed. Seven patients were treated with two therapy cycles, whereas 28 received three therapy cycles; one was treated with four therapy cycles, and only two patients received five therapy cycles. Median Gleason Score was 8 (range 6–10). RLT was performed in 24/38 (63%) of the patients before chemotherapy and in 14/38 (37%) after chemotherapy (**Tables 1** and **2**). 34/38 patients had high tumor burden (>10 lesions).

**Table 1:** Patients’ Characteristic.

Parameter	PR (n/%)	SD (n/%)	PD (n/%)	Total
**Age**				
<60	1	1	1	**3**
>60	8	19	8	**35**
**Chemotherapy**				
Prior	3	15	6	**24**
After	6	5	3	**14**
**Gleason Score**				
6-8	6	2	4	**12**
9-10	9	4	3	**16**

**Table 2:** showing patient’s age, Gleason Score (GS), number (No.) of radioligand therapy (RLT) cycles, prior enzalutamide therapy (enzalutamide), prior chemotherapy (Chemo), baseline PSA (PSAb), % PSA change from baseline, response o PSMA PET/CT.

	Age	GS	No. RLT	Tumor extent	Enza	Chemo	PSAb	% PSA Change	PET/CT Response
1	68	7	5	High	No	No	88,5	−92,35	PR
2	61	7	3	Low	No	No	93,21	464,96	PD
3	72	10	3	High	Yes	Yes	760,2	−50,41	PR
4	77		4	High	No	No	46,43	−4,59	PR
5	56	10	3	High	No	No	138,5	−98,23	PR
6	68	7	3	High	No	Yes	34,96	655,15	PD
7	71		2	Low	No	Yes	34,75	175,57	PD
8	64	9	3	Low	No	No	0,91	258,24	PR
9	51	8	3	High	No	No	12,14	−12,93	SD
10	75		3	High	Yes	No	4,53	38,63	PR
11	71	9	3	High	Yes	No	41,65	−65,52	PR
12	73	9	2	High	No	No	27,82	−84,15	PR
13	63		3	Low	Yes	No	3,9	−38,46	SD
14	82	9	3	High	No	No	1,79	−41,34	SD
15	65	9	2	High	No	Yes	199,2	72,69	PD
16	75	8	3	High	No	No	1,64	259,15	PR
17	73	8	2	High	Yes	Yes	1818	22,16	PD
18	77	9	3	High	No	No	24,25	28,45	PR
19	86	6	3	High	Yes	No	42,85	−96,06	PR
20	63	10	3	High	No	No	115,9	27,18	PR
21	65	NA	3	High	Yes	Yes	109	−29,91	PR
22	67	7	3	High	No	No	55,41	46	PR
23	76	NA	3	High	No	No	208,8	26,92	PR
24	73	NA	3	High	No	Yes	23,3	−46,09	PR
25	67	NA	2	High	No	Yes	334,9	428,81	PD
26	67	9	2	High	Yes	No	327	348,62	PD
27	72	7	5	High	Yes	No	46,8	167,09	PR
28	61	9	3	High	No	No	40,3	−60,55	SD
29	60	9	3	High	No	No	17,9	−74,3	PR
30	65	9	3	High	Yes	No	3,17	10,09	SD
31	69	9	3	High	No	No	745	24,3	PD
32	78	9	3	High	No	Yes	7,88	−4,44	SD
33	69	9	2	High	No	Yes	12,2	34,43	PR
34	52	7	3	High	No	Yes	21,3	351,17	PD
35	66	NA	3	High	Yes	Yes	186	−5,91	SD
36	74	7	3	High	No	Yes	244	−67,91	PR
37	87		3	High	Yes	No	110	−62,73	SD
38	61	7	3	High	No	Yes	21,3	−26,76	SD

PR, partial remission; SD, stable disease; PD, progressive disease; NA, not available; High, >10 lesions; low, ≤10 lesions.

### PSA Response

**Figure 1:**
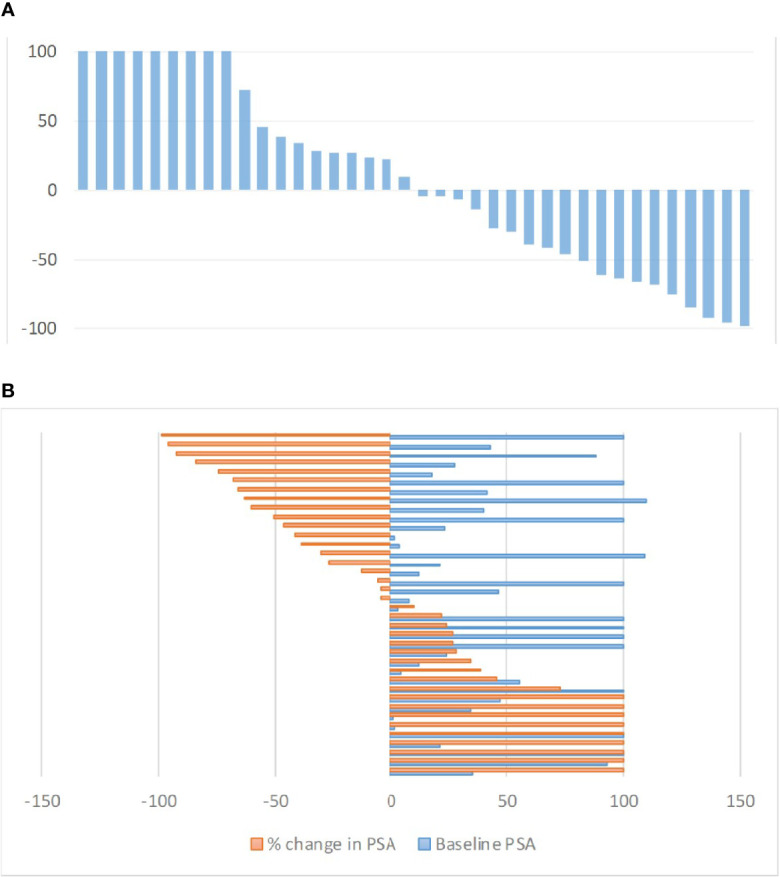
**(A)** Waterfall plot of maximum PSA changes (%) from baseline after two RLT cycles. PSA increase over 100% were cropped for ease of presentation. **(B)** Baseline PSA *vs.* maximum PSA changes (%) from baseline after two RLT cycles. Values over 100% were cropped for ease of presentation.

After two RLT cycles, 9/38 (23.7%) patients achieved PR; in 19/38 (50%) disease was stabilized (SD), whereas in 10/38 (26.3%) of patients disease was progressive (PD). **Figure 1A** shows the waterfall plot of the % change in PSA after two cycles of RLT. **Figure 1B** shows the max % changes in PSA in comparison to the baseline.

### PSMA PET/CT Response

Based on ^68^Ga-PSMA PET/CT response criteria, 20/38 (52.6%) of patients achieved PR after two RLT cycles; in 9/38 (23.7%) disease was stabilized (SD), whereas in 23.7% disease progressed (PD). The strength of agreement between PSA based response and PSMA PET/CT based criteria was only fair (kappa = 0.346).

Majority of the patients (five; 71%) treated with only two therapy cycles were previously managed with chemotherapy. In comparison only nine out of 31 patients (29%) receiving more than two therapy cycles were previously treated with chemotherapy. However, there was no significant association between disease status after two RLT cycles and pre-RLT chemotherapy (p = 0.127) or number of therapy cycles (two or more) and pre-RLT chemotherapy (p = 0.08).

### Survival

**Figure 2:**
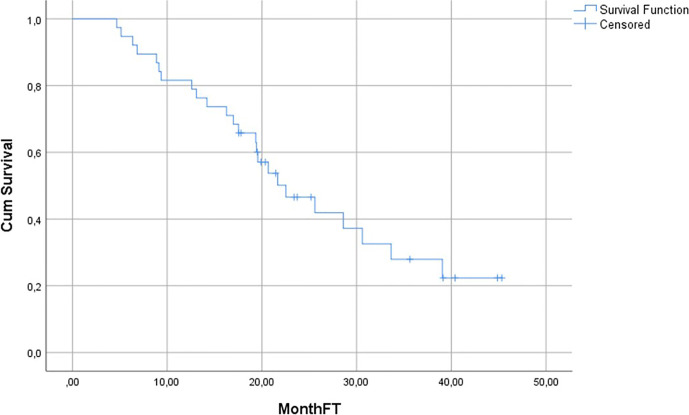
Overall Survival Kaplan–Meier Curve of the whole cohort.

**Figure 3:**
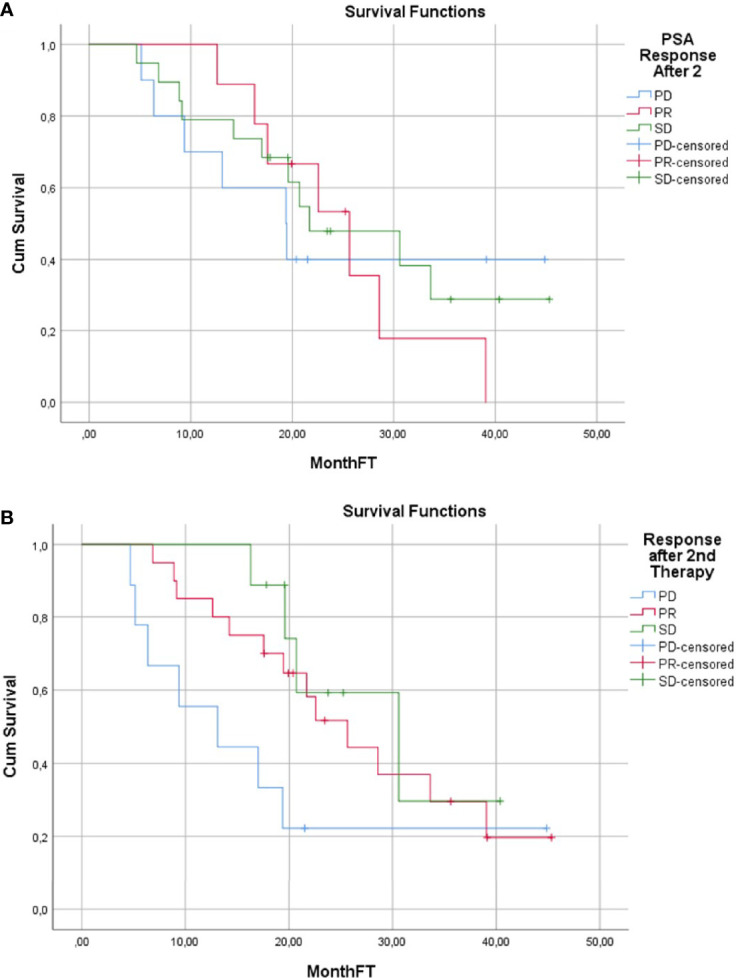
**(A)** Overall Survival Kaplan–Meier Curve based on PSMA PET/CT response evaluation. **(B)** Overall Survival Kaplan–Meier Curve based on PSA response evaluation.

**Figure 4:**
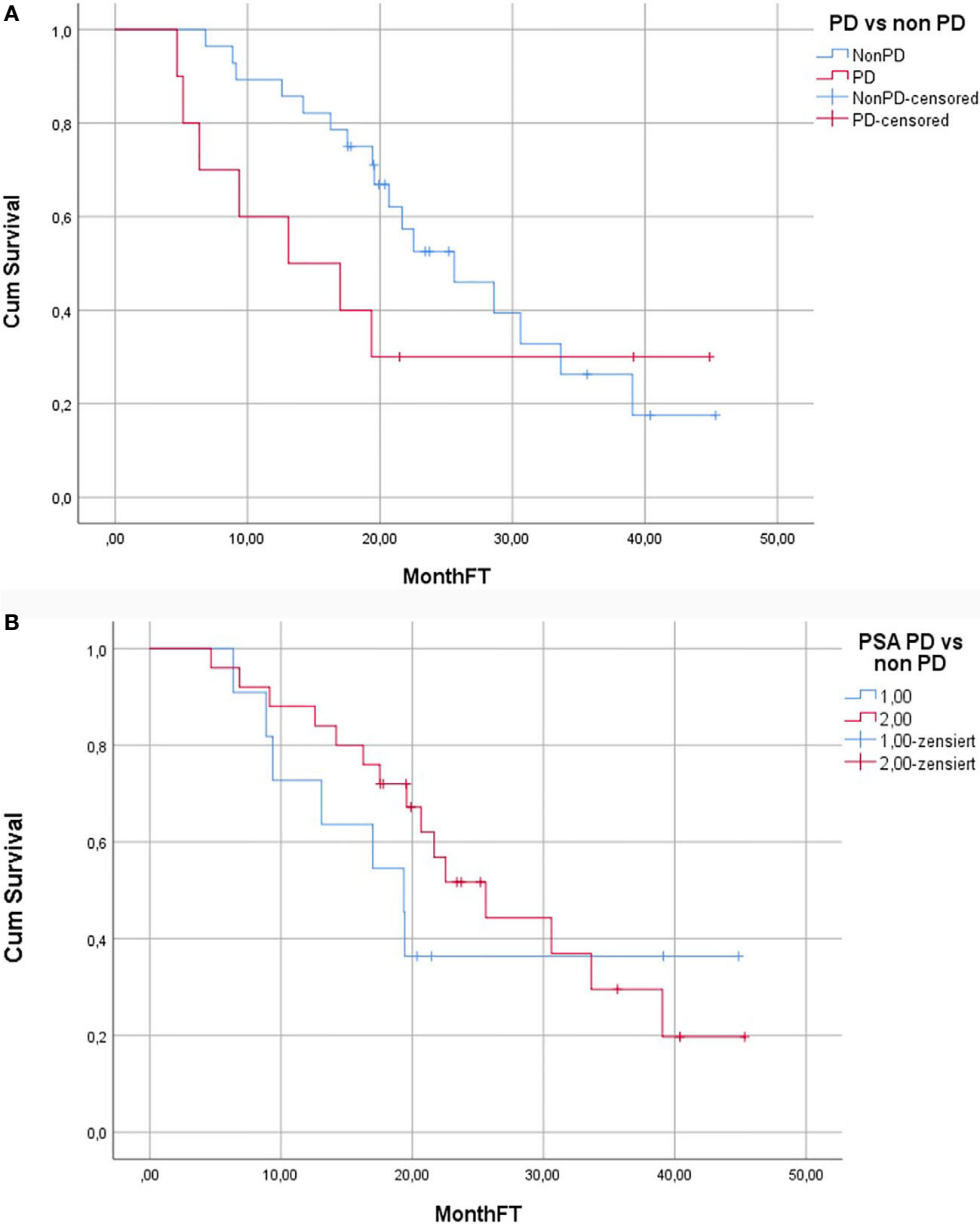
**(A)** Kaplan–Meier curve showing overall survival of patients with and without PD according to the PSMA PET/CT response. **(B)** Kaplan–Meier curve showing overall survival of patients with and without PD according to the PSA response.

Median duration of follow-up was 19.7 months (4.7–45.3). 24/38 (63%) patients died during the follow-up. Median OS was 22.5 months (95% CI: 15.8–29.2) (**Figure 2**). Median OS stratified to PSA response criteria was as follows: for patients classified as PR 25.6 months, SD 21.7 months and PD 19.4 months (p = 0.496); **Figure 3A**. Median OS stratified according to interim PSMA PET/CT response was as follows: PR 25.6 months, SD 30.6 months, PD 13.1 months (p = 0.013); **Figure 3B**. Median OS of patient with SD and PR clubbed together as non-PD was 25.6 months in comparison to median OS of 13.1 months for PD patients (p = 0.03). Patients with SD or PR on PSA response after two cycles was found to be 18.5 months in comparison to 12.5 months for patient having PD (p = 0.528). **Figure 4A** shows the median OS stratified according to PD *vs* non-PD based on PSMA PET/CT. **Figure 4B** shows the median OS in PD *vs* non-PD based on PSA response.

## Discussion

Robust and reliable surrogate markers for prediction of overall survival of a patient undergoing treatment with RLT is crucial, as not all PCa patients benefit from it. In this study we could demonstrate that interim RLT-REC-PCA after the 2^nd^ cycle is predictive of overall survival and is better suited for distinguishing PD from non-in comparison to PSA (**Figures 2A, B**). In our study, we observed biochemical response rates, as defined by a PSA decline ≥50%, in 23.5% of patients after two cycles of RLT. This result is somewhat different in comparison to other published studies (8, 18). Kambiz et al. have reported a PSA decline of ≥50% in 57% of the patients after 2^nd^ RLT, whereas Fendler et al. reported a PSA decline of ≥30% in 47% of patients (8, 18). This discrepancy may be due to the differences in the timing of PSA measurement used for biochemical response evaluation. Prostate cancer working group criteria suggest that PSA values at 12 weeks should be considered for response assessment with PSA (16). PSA measurement for response assessment in our study was performed at 8–10 weeks after therapy and, thus, closer to the recommended time point in comparison to other published studies or guidelines whereas in other studies any PSA values after the 2^nd^ treatment cycle were considered (8, 10, 11).

**Figure 5:**
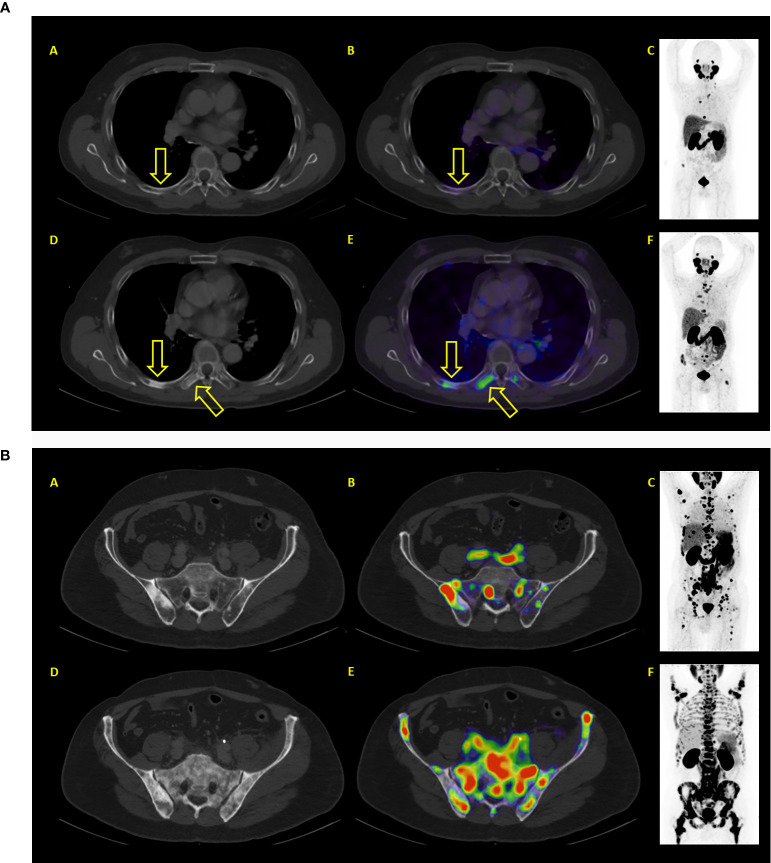
**(A)** Example of a patient where both PSA and PET showed similar results: progressive disease. After two therapy cycles PSA values increased from 93.2 to 214.2 ng/ml. PET/CT showed new bone lesions as well progression of previously known lesions. (A–C) represent baseline images whereas (C–E) represent interim PET/CT. (A, D): axial CT slices; (E): axial fused PET/CT images; (C, F) maximum intensity projection images. **(B)** Example of a patient where both PSA and PET showed similar results: progressive disease. After two therapy cycles PSA values increased from 327 to 1467 ng/ml. PET/CT showed diffuse new bone lesions. (A–C) represent baseline images whereas (C–E) represent interim PET/CT. (A, D): axial CT slices; (B, E): axial fused PET/CT images; (C, F) maximum intensity projection images.

**Figure 6:**
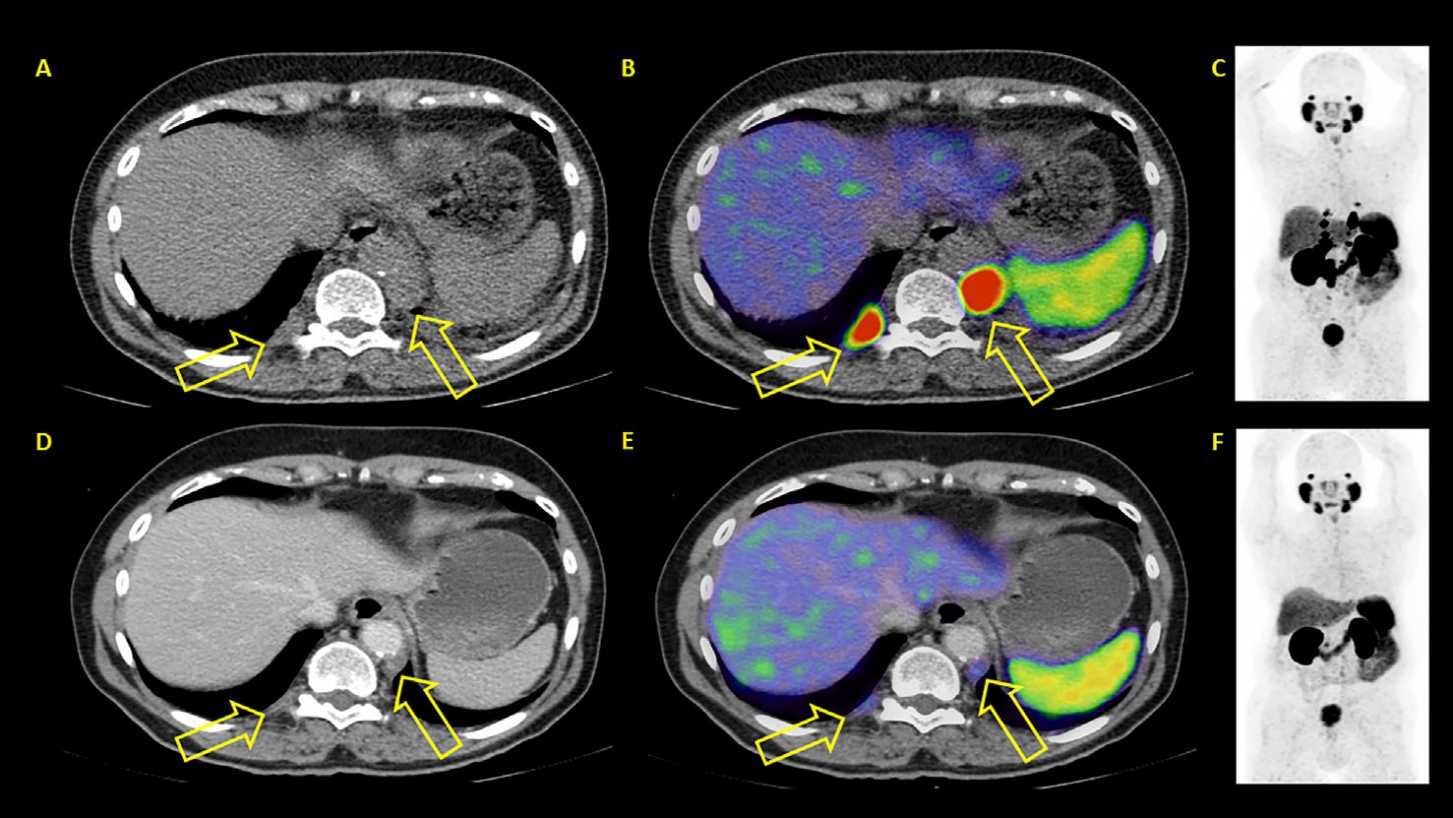
Example of a patient where both PSA and PET showed similar results: partial remission. After two therapy cycles PSA values decreased from 46.4 to 1.42 ng/ml. PET/CT also showed partial remission. **(A–C)** represent baseline images whereas **(D–F)** represent interim PET/CT. **(A, D)**: axial CT slices; **(B, E)**: axial fused PET/CT images; **(C, F)**: maximum intensity projection images.

**Figure 7:**
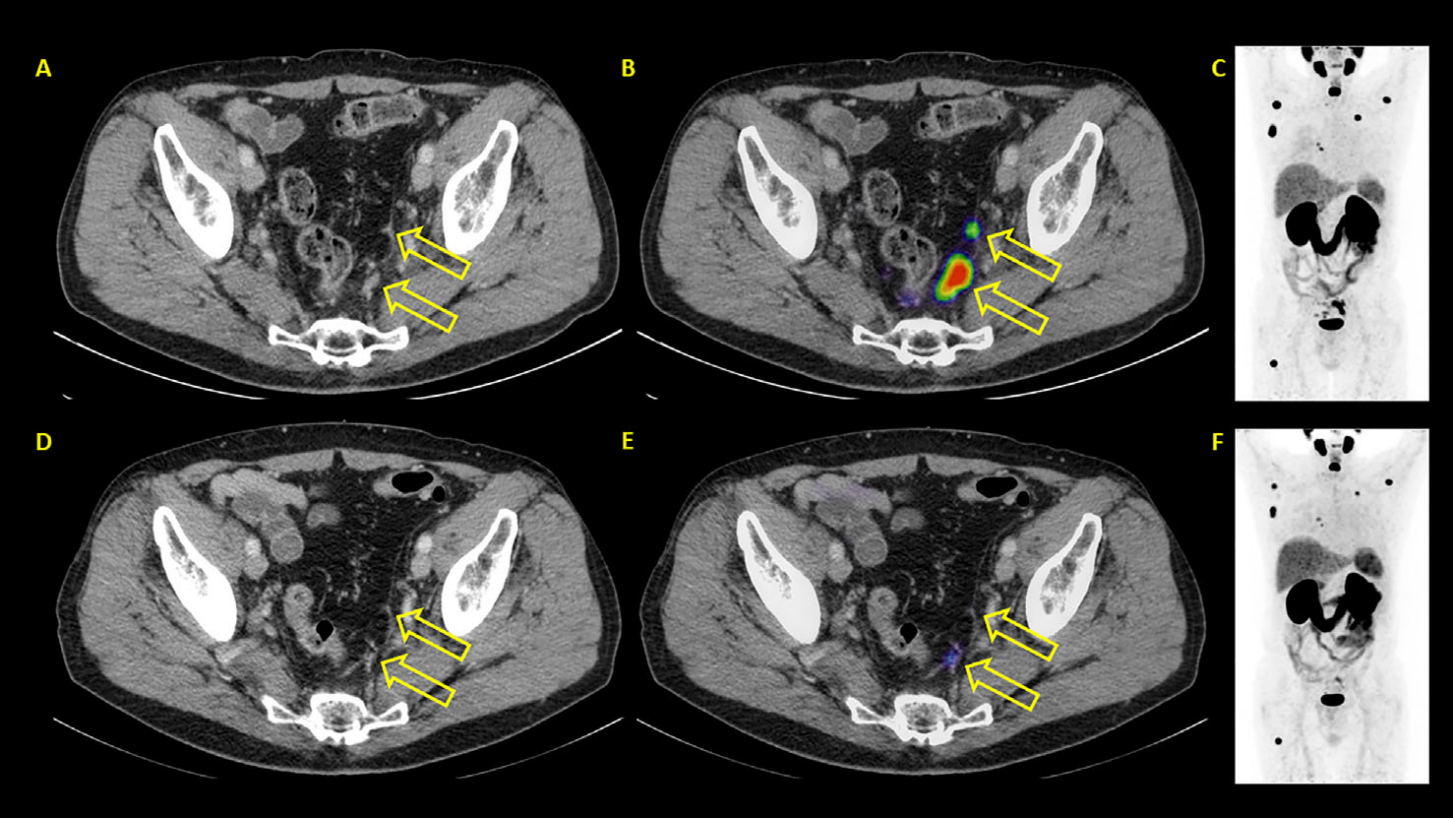
Example of a patient where PSA and PET showed discordant results. PSA showed progressive disease with PSA rising from 24.3 to 35.8 ng/ml. PSMA PET/CT showed partial remission. **(A–C)** represent baseline images whereas **(D–F)** represent interim PET/CT. **(A, D)**: axial CT slices; **(B, E)**: axial fused PET/CT images; **(C, F)**: maximum intensity projection images.

In contrast to PSA response, PSMA PET/CT based response evaluation showed objective response (52.6% PR and 23.7% SD) in about three-fourths of the patients and PD in 23.7%. **Figures 5**–**7** show examples of patients showing concordant and discordant results between PSA and RLT-REC-PCA response. Fendler et al. reported similar results with PR in 27%, SD in 40% (objective response 67%) and PD in 33% of patients based on ^68^Ga-PSMA PET/CT response assessment in 15 patients after two treatment cycles (19). A direct comparison of our results with previously published data is hampered by the fact that our image based response analysis was based upon RECIST 1.1 with integration PET data for bone metastases assessment whereas Fendler et al. used only measurable CT based assessment excluding bone lesions (16).

Baum et al. published results of molecular imaging based response evaluation in 25 patients (20); 56% showed PR in ^68^Ga-PSMA PET, whereas RECIST based evaluation showed partial remission in only 20% of the patients. The authors explained this discrepancy between molecular imaging and CT based response by the lower sensitivity of stand-alone CT in the assessment of skeletal lesions. Another difference in response evaluation to our study is that we performed ^68^Ga-PSMA PET/CT 8 weeks after the 2^nd^ cycle of RLT, and thereafter response evaluation was based on a standard procedure, whereas Baum et al. performed response evaluation 6 months after the 2^nd^ cycle of RLT (20).

It has been shown that post therapy PSA values are significantly influenced by the release of PSA from tumor cells responding to treatment. This makes PSA an unreliable biomarker in the context of clinical management, and more often than not in case of clinical and biochemical discrepancy, imaging is called upon to assess the response. Interestingly, there was only a weak correlation between these two response evaluation methods. The limitation of PSA as a biomarker for response assessment has been investigated and reported several times (18). It must be mentioned here that there are certain other PSA based parameters which needs further evaluation *e.g.* initial PSA, PSA nadir, and PSA kinetic.

To resolve the question which one of the two tools, PSA or PSMA PET/CT, is more appropriate for response assessment, we also analyzed the predictive value of interim PSMA PET/CT and PSA on OS. Median survival of patients with progressive disease after two RLT is 1.93 times shorter in comparison to patients without PD. In comparison, PSA was not found to be predictive for OS; we also could not observe any differences in the OS between PD and non-PD patients. It remains to be seen in larger study cohorts if the interim PET performed 8–10 weeks after the 2^nd^ cycle of therapy is the best time point for response assessment. Interestingly, the median OS of patients achieving PR on RLT-REC-PCA was not significantly different as compared to patients achieving SD. One plausible explanation for this paradigm is that RLT-REC-PCA has RECIST 1.1 as its base and includes PET component only to diagnose or characterize new lesions or bone lesions and does not take into consideration the changes in PSMA expression on target lesions. However, several studies have shown that the PSMA expression on PCa metastases is variable in time (temporal heterogeneity) and location of tumors (spatial heterogeneity) (21). For example, liver metastases have shown lower PSMA expression in comparison to other sites of metastases. On the contrary, lymph nodes generally show homogeneous PSMA expression. Bone/bone marrow metastases can also have variable PSMA expression (22). This often necessitates including CT information in PET evaluation for correct assessment of tumor response.

## Limitations and Future Perspective

Limitations of this explorative study are the retrospective approach and the relatively low and heterogeneous patient population. It remains to be seen if these results maintain their significance in larger patient population. However, these limitations are somewhat overcome by a relatively long median follow-up of approximately 20 months. Future studies are needed to find out the ways of including more PET information in RLT-REC-PCA *e.g.* PSA kinetic, PSMA heterogeneity, total PSMA avid lesions volume *etc*.

## Conclusions

Interim PSMA PET/CT performed at 8–10 weeks after the 2^nd^ therapy cycle is predictive of overall survival and PD of patients treated with ^177^Lu-PSMA. In contrast, PSA appears to have only limited predictive value.

## Data Availability Statement

Major part of the data is already included in the tables. For further data please contact authors.

## Ethics Statement

The retrospective analysis was performed in accordance with the ethical standards of the institutional ethics committee and with the 1964 Helsinki declaration and its later amendments. All patients analyzed in this study gave written informed consent for treatment and analyses of their clinical data for research purposes being aware that treatment with 177Lu-PSMA and imaging with 68Ga-PSMA were both performed under national regulations of compassionate use of a non-approved drug according to AMG §13. 2b (15). All patients suffered from confirmed disease progression based on PSA in a metastasized stage showing PSMA expression on 68Ga-PSMA PET/CT. The decision for conducting the treatment with 177Lu PSMA was established in an interdisciplinary setting. The local institutional ethics committee (EA2/177/17) approved the publication of data.

## Author Contributions

VP and WB were involved in the study design, management of patients, writing and reading the manuscript, and statistical analyses. KH was involved in the management of patients, writing, and reading the manuscript. MM as a radiologist and nuclear medicine physician was involved in PET/CT analyses (together with VP and KH), writing, and reading the manuscript. SP was involved in radiolabeling of ^68^Ga-PSMA and ^177^Lu-PSMA, reading, and writing the manuscript. NC was involved in patient management, reading, and writing the manuscript. All authors contributed to the article and approved the submitted version.

## Conflict of Interest

The authors declare that the research was conducted in the absence of any commercial or financial relationships that could be construed as a potential conflict of interest.

## References

[1] Siegel RL Miller KD Jemal A. Cancer statistics, 2019. CA Cancer J Clin (2019) 69:7–34. 10.3322/caac.21551 30620402

[2] Ghosh A Heston WD. Tumor target prostate specific membrane antigen (PSMA) and its regulation in prostate cancer. J Cell Biochem (2004) 91:528–39. 10.1002/jcb.10661 14755683

[3] Bouchelouche K Choyke PL Capala J. Prostate specific membrane antigen- a target for imaging and therapy with radionuclides. Discov Med (2010) 9:55–61. 20102687 PMC3410553

[4] Afshar-Oromieh A Malcher A Eder M Eisenhut M Linhart HG Hadaschik BA. PET imaging with a [68Ga]gallium-labelled PSMA ligand for the diagnosis of prostate cancer: biodistribution in humans and first evaluation of tumour lesions. Eur J Nucl Med Mol Imaging (2013) 40:486–95. 10.1007/s00259-012-2298-2 23179945

[5] Prasad V Steffen IG Diederichs G Makowski MR Wust P Brenner W. Biodistribution of [(68)Ga]PSMA-HBED-CC in Patients with Prostate Cancer: Characterization of Uptake in Normal Organs and Tumour Lesions. Mol Imaging Biol (2016) 18:428–36. 10.1007/s11307-016-0945-x 27038316

[6] Ahmadzadehfar H Rahbar K Kurpig S Bogemann M Claesener M Eppard E. Early side effects and first results of radioligand therapy with (177)Lu-DKFZ-617 PSMA of castrate-resistant metastatic prostate cancer: a two-centre study. EJNMMI Res (2015) 5:114. 10.1186/s13550-015-0114-2 26099227 PMC4477007

[7] Weineisen M Schottelius M Simecek J Baum RP Yildiz A Beykan S. 68Ga- and 177Lu-Labeled PSMA I&T: Optimization of a PSMA-Targeted Theranostic Concept and First Proof-of-Concept Human Studies. J Nucl Med (2015) 56:1169–76. 10.2967/jnumed.115.158550 26089548

[8] Rahbar K Ahmadzadehfar H Kratochwil C Haberkorn U Schafers M Essler M. German Multicenter Study Investigating 177Lu-PSMA-617 Radioligand Therapy in Advanced Prostate Cancer Patients. J Nucl Med (2017) 58:85–90. 10.2967/jnumed.116.183194 27765862

[9] Hofman MS Violet J Hicks RJ Ferdinandus J Thang SP Akhurst T. [(177)Lu]-PSMA-617 radionuclide treatment in patients with metastatic castration-resistant prostate cancer (LuPSMA trial): a single-centre, single-arm, phase 2 study. Lancet Oncol (2018) 19:825–33. 10.1016/S1470-2045(18)30198-0 29752180

[10] Fendler WP Kratochwil C Ahmadzadehfar H Rahbar K Baum RP Schmidt M. [177Lu-PSMA-617 therapy, dosimetry and follow-up in patients with metastatic castration-resistant prostate cancer]. Nuklearmedizin (2016) 55:123–8. 10.1055/s-00034924 27350005

[11] Kratochwil C Fendler WP Eiber M Baum R Bozkurt MF Czernin J. EANM procedure guidelines for radionuclide therapy with (177)Lu-labelled PSMA-ligands ((177)Lu-PSMA-RLT). Eur J Nucl Med Mol Imaging (2019) 46:2536–44. 10.1007/s00259-019-04485-3 31440799

[12] Heck MM Tauber R Schwaiger S Retz M D’Alessandria C Maurer T. Treatment Outcome, Toxicity, and Predictive Factors for Radioligand Therapy with (177)Lu-PSMA-I&T in Metastatic Castration-resistant Prostate Cancer. Eur Urol (2018) 75:920–6. 10.1016/j.eururo.2018.11.016 30473431

[13] Emmett L Crumbaker M Ho B Willowson K Eu P Ratnayake L. Results of a Prospective Phase 2 Pilot Trial of (177)Lu-PSMA-617 Therapy for Metastatic Castration-Resistant Prostate Cancer Including Imaging Predictors of Treatment Response and Patterns of Progression. Clin Genitourin Cancer (2019) 17:15–22. 10.1016/j.clgc.2018.09.014 30425003

[14] Gesetz über den Verkehr mit arzneimitteln (arzneimittelgesetz - aMG). Available at: https://www.gesetze-im-internet.de/amg_1976/index.html. (letzter Zugriff: 20. September 2018). Stand: 18. Juli 2017.

[15] Kulkarni HR Singh A Schuchardt C Niepsch K Sayeg M Leshch Y. PSMA-Based Radioligand Therapy for Metastatic Castration-Resistant Prostate Cancer: The Bad Berka Experience Since 2013. J Nucl Med (2016) 57:97S–104S. 10.2967/jnumed.115.170167 27694180

[16] Scher HI Halabi S Tannock I Morris M Sternberg CN Carducci MA. Design and end points of clinical trials for patients with progressive prostate cancer and castrate levels of testosterone: recommendations of the Prostate Cancer Clinical Trials Working Group. J Clin Oncol (2008) 26:1148–59. 10.1200/JCO.2007.12.4487 PMC4010133 18309951

[17] Janssen JC Meissner S Woythal N Prasad V Brenner W Diederichs G. Comparison of hybrid (68)Ga-PSMA-PET/CT and (99m)Tc-DPD-SPECT/CT for the detection of bone metastases in prostate cancer patients: Additional value of morphologic information from low dose CT. Eur Radiol (2018) 28:610–9. 10.1007/s00330-017-4994-6 28779400

[18] Paximadis P Najy AJ Snyder M Kim HR. The interaction between androgen receptor and PDGF-D in the radiation response of prostate carcinoma. Prostate (2016) 76:534–42. 10.1002/pros.23135 PMC6864751 26732854

[19] Fendler WP Reinhardt S Ilhan H Delker A Boning G Gildehaus FJ. Preliminary experience with dosimetry, response and patient reported outcome after 177Lu-PSMA-617 therapy for metastatic castration-resistant prostate cancer. Oncotarget (2017) 8:3581–90. 10.18632/oncotarget.12240 PMC5356905 27683041

[20] Baum RP Kulkarni HR Schuchardt C Singh A Wirtz M Wiessalla S. 177Lu-Labeled Prostate-Specific Membrane Antigen Radioligand Therapy of Metastatic Castration-Resistant Prostate Cancer: Safety and Efficacy. J Nucl Med (2016) 57:1006–13. 10.2967/jnumed.115.168443 26795286

[21] Miyahira AK Pienta KJ Morris MJ Bander NH Baum RP Fendler WP. Meeting report from the Prostate Cancer Foundation PSMA-directed radionuclide scientific working group. Prostate (2018) 78:775–89. 10.1002/pros.23642 29717499

[22] Khurshid Z Ahmadzadehfar H Gaertner FC Papp L Zsoter N Essler M. Role of textural heterogeneity parameters in patient selection for 177Lu-PSMA therapy via response prediction. Oncotarget (2018) 9:33312–21. 10.18632/oncotarget.26051 PMC6161784 30279962

